# Probiotic-infused activated charcoal/ hydroxyapatite microbeads: a novel strategy to disinfection

**DOI:** 10.7717/peerj.20803

**Published:** 2026-02-18

**Authors:** Mohammed A. Alqumber

**Affiliations:** Laboratory Medicine Department, Faculty of Applied Medical Sciences, Alaqiq, Al-Baha, Saudi Arabia

**Keywords:** Probiotic disinfectant, Activated charcoal microbeads, Hydroxyapatite microbeads, Competitive exclusion, Antimicrobial biofilm, Pathogen suppression, Ecological disinfection, Surface decontamination, Sustainable disinfection

## Abstract

**Objectives:**

To develop a non-toxic, probiotic-infused activated charcoal/hydroxyapatite microbeads (PIMD) formulated with activated charcoal and hydroxyapatite. The formulation is designed to establish a stable probiotic biofilm on high-risk surfaces, such as medical sink basins and food cutting boards. Its dual mechanism aims to achieve rapid pathogen inhibition and long-term protection through sustained competitive exclusion, including against antibiotic-resistant microorganisms.

**Methods:**

An applied study was conducted in the Al-Baha region of Saudi Arabia (January 2021–May 2025) employing probiotic-based biotechnology to engineer spatially segregated microenvironments using activated charcoal–hydroxyapatite microbeads. The study integrated ecological modeling concepts—including Gause’s competitive exclusion principle, Lotka–Volterra dynamics and agent-based cross-feeding simulations—with antimicrobial sensitivity assays and surface disinfection trials. Twelve probiotic strains capable of competitively excluding pathogens and producing antimicrobial compounds were encapsulated within activated charcoal and hydroxyapatite porous microbeads. The efficacy of the novel disinfectant was evaluated on stainless steel sink basins, food-grade cutting boards, and culture plates challenged with 43 pathogenic strains.

**Results:**

The PIMD formulation remained physically stable under storage conditions, with probiotic viability largely preserved at –20 °C (0.46 log_10_ CFU/mL reduction) and 4 °C (0.89 log_10_ reduction) over 365 days. PIMD exhibited rapid broad-spectrum disinfection, reducing pathogen loads by ≥ 99.9% within 1 h, and maintained protective biofilm-mediated probiotic viability at ≥ 5.0 log_10_ CFU/cm^2^ for at least 21 days post-application across tested surfaces, including cutting boards, sink basins, and outdoor tiles. The mean inhibition zone diameter across 43 pathogens was 13.84 ± 1.23 mm, with a bactericidal outcome observed for 34.9% of tested strains.

**Conclusion:**

PIMD represents a dual-action strategy combining rapid disinfection with sustainable microbial balance, for reducing reliance on antibiotics and chemical disinfectants while enhancing surface safety in diverse environments such as healthcare, food facilities, and beyond.

## Introduction

The persistent threat of pathogenic microorganisms in various environments necessitates innovative disinfection strategies (Salam et al., 2023; Murray & Collaborators, 2024). Conventional methods of disinfection and antisepsis suffer from enduring limitations, notably the development of resistance, variable efficacy against pathogens, environmental impact, disruption of beneficial microbial communities and inherent toxicity risks, such as irritant and allergic contact dermatitis (ICD) (Jing et al., 2020; Maillard & Pascoe, 2024). Additionally, they pose potential threats to fertility and cancer risk (Lanrewaju et al., 2022; Kirkpatrick, Melin & Hrubec, 2025). Moreover, the implementation of regular antimicrobial cleaning and the widespread use of antibiotics and chemical disinfectants to address contaminations, has contributed to the emergence of drug-resistant pathogens, resulting in a crisis (Salam et al., 2023; Ranjbar & Alam, 2023). Furthermore, the indiscriminate action of chemical disinfectants disrupts microbial ecosystems, reducing diversity through the elimination of both harmful and beneficial bacteria—a phenomenon termed microbial dysbiosis (Ramakrishna & Patankar, 2023; Zhang & Lu, 2023). This dysbiosis creates vacant niches that favor pathogen colonization (Stone et al., 2020), as observed in high-risk surfaces like healthcare sink drains (Anantharajah et al., 2024; Volling et al., 2024) and food cutting boards (Dantas et al., 2018; Tasanapak et al., 2023). These surfaces, with their microabrasions and cracks, provide ideal niches for pathogen adhesion and biofilm maturation (Anantharajah et al., 2024; Ahnrud et al., 2018). Consequently, this poses substantial health hazards, and conventional disinfection approaches often prove inadequate, leading to persistent contamination and infection outbreaks over extended periods (Lai et al., 2023; Inkster, 2024). Enhanced disinfection techniques are urgently needed to address persistent pathogen growth on surfaces, and to prevent microbial imbalances (dysbiosis) and diseases. To counteract dysbiosis, ecological strategies such as competitive exclusion offer a targeted alternative. Moreover, Gause’s Law, also known as the Competitive Exclusion Principle, supports the importance of probiotics in maintaining a healthy microbial balance (Stone et al., 2020). Formulated by the Soviet ecologist Gause (1934), Gause’s Law indicates that two species vying for the same limited resources cannot coexist indefinitely; and one species will inevitably outcompete and eliminate the other, resulting in the exclusion of the less fit species from that specific ecological niche. Here, we propose a probiotic-based disinfection strategy to address these gaps by introducing and testing a novel Probiotic-Infused Microbead Disinfectant (PIMD), which unlike prior approaches, uniquely combines a consortium of highly diverse beneficial probiotics, that include antimicrobial-producing strains, with a protective matrix of shielding microbeads, made of activated charcoal and hydroxyapatite beads, to enhance the disinfection efficacy, duration and environmental safety. To support this ecological framework, we incorporated computational modeling tools—specifically, Lotka–Volterra dynamics (Qian, Lan & Venturelli, 2021; Van den Berg et al., 2022), and agent-based cross-feeding simulations (Aminian-Dehkordi et al., 2025)—to predict and optimize microbial interactions within the PIMD matrix. These models allowed us to simulate how probiotic strains interact with one another and with pathogens over time and space, offering theoretical validation of competitive exclusion, cooperative metabolite exchange, and niche dominance within surface biofilms. We hypothesize that PIMD will overcome the limitations of conventional disinfectants by combining immediate antimicrobial activity (*via* bacteriocins, lipopeptides) with long-term biofilm-mediated exclusion, enabled by activated charcoal and hydroxyapatite. That is, the incorporation of probiotics, acknowledged for their health benefits, offers a promising avenue to address microbial imbalances, prevent disease, and foster a healthier environment (Yadav et al., 2022; Tomé et al., 2023). It is already reported that beneficial microbes can maintain overall health and reduce the incidence of harmful pathogens (Martyniak et al., 2021; Todorov, Tagg & Ivanova, 2021). Moreover, a recent study indicated that disinfectants strongly promote the survival of deposited pathogens on surfaces after the disinfection, whereas probiotic cleaners, particularly those with diverse microbial strains, show efficacy in forming stable surface biofilms, thus excluding pathogens (Stone et al., 2020). It is well established that charcoal supports prolific bacterial growth (Gorelick, Mead & Kelly, 1951; Bolan et al., 2023), and hydroxyapatite facilitates both bacterial colonization and biofilm development (Moon, Seo & Kwon, 2025). This ecological partitioning mirrors multispecies biofilm succession and spatial distribution of multispecies within biofilms, allowing microbial heterogeneity to enhance community resilience (Yao et al., 2022). Motivated by these properties—alongside our previous findings on competitive exclusion in bovine microbiota (Al-Qumber & Tagg, 2006) and the persistence of pathogens on high-risk surfaces (Alqumber, 2014), we developed PIMD to possess rapid antimicrobial action through probiotic-derived antimicrobials, while ensuring sustained exclusion of pathogens *via* biofilm-mediated protection. This dual-action strategy was herein successfully tested on food cutting boards, sinks and outdoor tiles contaminated with pathogens from non-human primates (Alqumber, 2024).

## Materials & Methods

### Study design

**Figure 1:**
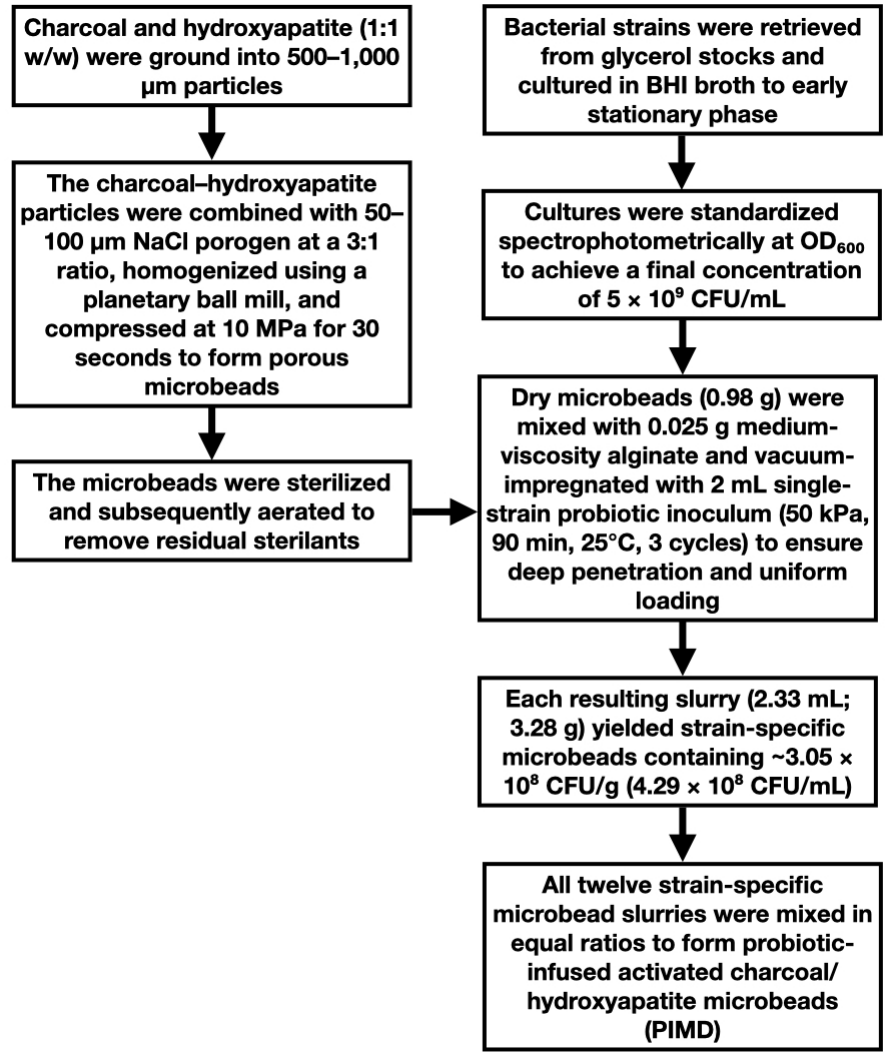
Workflow diagram for the development, fabrication, and validation of the Probiotic-Infused Microbead Disinfectant (PIMD). Stepwise workflow used to develop the Probiotic-Infused Microbead Disinfectant (PIMD) from charcoal–hydroxyapatite microbeads and standardized bacterial inoculants grown in brain heart infusion (BHI) broth.

This applied study was conducted in Al-Baha, Saudi Arabia (January 2021–May 2025) to develop and evaluate a probiotic-infused microbead disinfectant (PIMD) through interdisciplinary biotechnology approaches, including material engineering, microbial cultivation, and *in vitro* efficacy testing. A systematic review of peer-reviewed articles was conducted using PubMed (Medline) and EMBASE databases. The literature search employed key terms including probiotic, probiotic disinfectant, competitive exclusion and pathogens, activated charcoal and biofilm, activated charcoal and bacterial growth, hydroxyapatite and bacterial colonization, microbial dysbiosis, Lotka–Volterra and microbial competition, sink drain and pathogen persistence, cutting board and biofilm resistance, probiotic microbeads and encapsulation, and chemical disinfectant toxicity. These terms were selected to systematically capture interdisciplinary studies on ecological disinfection strategies, material-enabled probiotic delivery, and pathogen suppression mechanisms. Articles were screened for relevance to microbial and biofilm growth and resistance, surface disinfection challenges, and probiotic applications. After title/abstract screening, relevant full-text articles were retrieved and analyzed to inform the study design, probiotic selection, and material synthesis protocols. All chemicals and reagents were obtained from MilliporeSigma (St. Louis, MO, USA) unless otherwise stated. The experimental workflow, from bacterial inoculant preparation to Probiotic-Infused Microbead Disinfectant (PIMD) formulation, is summarized in Fig. 1, providing an overview of PIMD design and procedural steps.

### Bacterial inoculants

All bacterial inoculants were retrieved from the Al-Baha University Laboratory Medicine Department, Al-Baha, Saudi Arabia glycerol stocks and propagated to early stationary phase in brain heart infusion (BHI) broth (Thermo Fisher Scientific, Waltham, MA, USA) at 37 °C for 12 h. Aeration with 5% CO_2_ supplementation was used for all bacteria except *Campylobacter jejuni* (microaerophilic: 5% O_2_, 10% CO_2_, 85% N_2_) and *Bifidobacterium* spp. (anaerobic: 5% H_2_, 10% CO_2_, 85% N_2_). Next, bacterial cells were harvested by gentle centrifugation at 3,000× *g* for 15 min at 4 °C and resuspended in fresh BHI, followed by spectrophotometric standardization at an optical density of 600 nm to achieve final concentrations of 5 × 10^9^ colony-forming units per mL (CFU/mL) for probiotic strains and 1.5 × 10^8^ CFU/mL for indicator pathogens. For probiotic strains, the diluent BHI was supplemented with cryoprotective agents—5% (v/v) trehalose, 2% (v/v) 1,2-propanediol, 10% (v/v) glycerol, and 2% (v/v) dimethyl sulfoxide (DMSO) to preserve cell viability without altering target CFU concentrations, as previously described (Aarattuthodi et al., 2025).

### Vacuum-assisted probiotic encapsulation in porous charcoal- hydroxyapatite microbeads

The probiotic-shielding microbeads were fabricated by first grinding equal parts (1:1 w/w) of charcoal and hydroxyapatite into 500–1,000 µm particles using a Retsch BB 500 jaw crusher (Retsch GmbH, Haan, Germany). This composite powder was uniformly blended with 50–100 µm sieved NaCl porogen particles (50–100 µm, Thermo Fisher Scientific) at a 3:1 (w/w) matrix-to-porogen ratio using a planetary ball mill PM 100 (Retsch GmbH). The homogeneous mixture was subsequently compressed under 10 MPa uniaxial pressure for 30 s using a hydraulic press (Model 3850; Carver Inc.) equipped with stainless steel molds to form structurally stable porous microbeads. Interconnected pore networks were generated through controlled dissolution of the NaCl porogen in chilled sterile water (4 °C) under gentle orbital agitation (50 rpm, Jeio Tech IS-971R), for 2 h. For sterilization before probiotic impregnation, the microbeads were first exposed to chloroform vapor for 24 h, 25 °C. Afterward, the microbeads were exposed to hydrogen peroxide (H_2_O_2_) vapor for 6 h at 40 °C and underwent 70% ethanol wash, followed by aeration (14 days, 40 °C with 10 air changes/hour), to remove any residual chloroform, H_2_O_2_ or ethanol. After aeration, verification of sterilant removal was performed. Hydrogen peroxide was assayed using the potassium iodide–starch colorimetric method, wherein two mL of the microbeads were reacted with 0.2 mL of 1% (w/v) potassium iodide solution and 0.1 mL of freshly prepared 0.5% (w/v) soluble starch indicator (both from MilliporeSigma); the absence of blue coloration and the disappearance of any transient tint upon addition of a drop of catalase solution confirmed safe, non-detectable H_2_O_2_ levels (<1 mg/L). Ethanol residues were tested using the Rapid Response™ Alcohol Test Strip (Lochness Medical Supplies Inc.), which provides detection down to 0.02–0.3%; no observable color change confirmed ethanol concentrations below thresholds. Residual chloroform was quantified by purge-and-trap GC–MS (Agilent 7890/5977B; Agilent Technologies). A five mL aliquot of the bead aqueous extract was purged with helium (40 mL/min, 11 min, 40  °C), trapped on a dual-bed sorbent composed of Tenax TA [poly(2,6-diphenyl-p-phenylene oxide), 60/80 mesh] and silica gel (35/60 mesh) in a 2:1 w/w ratio, and thermally desorbed at 250 °C. Separation was achieved on a DB-624 column (30 m × 0.25 mm × 1.4 µm), and detection was performed in EI mode (70 eV, m/z 35–300). Calibration (0.1–100 µg/L) yielded correlation coefficient (R^2^) > 0.995, with a method detection limit of 0.2 µg/mL. No chloroform peaks were observed above the method detection limit, confirming effective aeration and sterilant removal. Afterward, 0.98 g (dry weight) aliquots were combined with 0.025 g medium viscosity alginate as a binding agent (Wang et al., 2024), to which two mL probiotic inoculum (single-strain), was vacuum-impregnated (50 kPa, 90 min, 25 °C, 3 cycles) using a GenVac MP2 system (NextGen Material Testing) to facilitate deep penetration and uniform adsorption of probiotics within the porous matrix, creating strain-specific microbeads in a homogeneous slurry with a total volume of 2.33 mL and a final mass of 3.28 g, harboring approximately 3.05 × 10^8^ CFU/g, corresponding to 4.29 × 10^8^ CFU/mL, based on volumetric, gravimetric and probiotic bacteria survival rate quantifications (method described below) of the final probiotic slurry. The probiotics used were: *Bacillus pumilus* strain 33 (Al-Qumber & Tagg, 2006), *Bacillus licheniformis* B63 (Hou et al., 2025), *Bacillus subtilis* BS50 (Garvey et al., 2022), *Lactobacillus acidophilus* DDS-1 (Vemuri et al., 2022), *Lactobacillus plantarum* SF9C (Butorac et al., 2020), *Lactobacillus helveticus* R0052 (De Oliveira et al., 2023), *Lacticaseibacillus rhamnosus* R0011 (Gupta et al., 2022), *Lacticaseibacillus rhamnosus* GR-1 (Petrova, Reid & Ter Haar, 2021), *Bifidobacterium animalis* subsp. *lactis* HN019 (Cheng, Laitila & Ouwehand, 2021), *Bifidobacterium infantis* 35624 (Roman et al., 2018), *Bifidobacterium bifidum* BGN4 (Ku et al., 2016), and *Pediococcus acidilactici* R1001 (Tremblay et al., 2021). Subsequently, the probiotics-infused microbeads corresponding to each strain were combined in equal ratios. This segregated impregnation strategy aims to allow oxygen-sensitive strains (*e.g.*, *Bifidobacterium*) and aerobic strains (*e.g.*, *Bacillus*) to colonize different microbeads. This spatial compartmentalization aims to mitigate cross-inhibition while enabling synergistic antimicrobial secretion (*e.g.*, bacteriocins, organic acids) into the shared carrier matrix.

### Microbead characterization for reproducibility

Following NaCl porogen dissolution, the microbeads were analyzed for pore architecture and particle size. Pore size distribution was determined *via* mercury intrusion porosimetry (Micromeritics AutoPore V), yielding an average pore diameter of 85–105 µm and a total porosity of 48 ± 3% (v/v). Probiotic loading efficiency was quantified by dissolving 50 beads in 10 mL sterile PBS with mild vortexing, followed by serial dilution and plating as described in the Probiotic Bacteria Survival Rate Quantifications section. The average loading reached 3.05 × 10^8^ CFU/g. All measurements were performed in triplicate across four independent production batches.

### Formulation of the Probiotic-Infused Microbead Disinfectant (PIMD)

First, a carrier matrix was prepared aseptically, consisting of the following polysaccharide gelling agent: 2.5% w/w prebiotic inulin fructooligosaccharide (Frutafit HD), 10% w/w sodium alginate (Loja Synth, Loja, Brazil), 4% w/w prebiotic low-methoxyl citric pectin (Plury Química, Brazil) (Yadav et al., 2022; Martyniak et al., 2021; Wang et al., 2024). Natural emulsifiers and adhesion-enhancing stabilizers—specifically 3.3 % w/w lecithin, 3.3 % w/w glycerol, 3.3 % w/w polysorbate, and 1 % w/w xanthan gum—were used to lower interfacial tension, improve dispersion, and promote stable adhesion to substrates (*e.g.*, stainless steel and polymeric cutting boards), thereby enhancing probiotic effects (Asase & Glukhareva, 2023; Zhuang, Clark & Acevedo, 2021; Ziar & Riazi, 2022; Van Holm et al., 2022). Additionally, 0.25% w/w muscone fragrance was included as a biocompatible antioxidant and odor-masking agent to neutralize organic substrate odors (Wang et al., 2025). The formulation was brought to a final mass of 1 kg with sterile phosphate-buffered saline (PBS; 137 mM NaCl, 2.7 mM KCl, 8 mM Na_2_HPO_4_, 2 mM KH_2_PO_4_, pH 7.4) to provide an isotonic, physiologically compatible medium for the probiotics. Next, 33 g of Al-Jouf olive oil (Jouf Agricultural Development Company, Al-Jouf Skaka, Saudi Arabia), 17 g of coconut oil (Bioriginal, Bosland, Netherlands), and 17 g of Awassi mutton tallow (sourced locally) were combined. Olive and coconut oils and mutton tallow were incorporated as a lipid matrix component providing antioxidant capacity and moisture retention. Their hydrophobic composition created a stable film that reduced desiccation stress and improved the formulation’s adhesion and persistence on applied surfaces. The lipid mixture was then homogenized with 12 g of trehalose, followed by the addition of 50 mL each of 1,2-propanediol (propylene glycol), glycerol, and DMSO as cryoprotectants (Aarattuthodi et al., 2025). The mixture was stirred continuously at 58 °C for 45 min until it achieved 7 Pascal-seconds (Pa s) consistency as measured by MSE PRO Digital Viscometer (MSE Supplies LLC, USA). Finally, equal volumes (1:1 v/v) of probiotic microbead slurry and carrier matrix were mixed to form a homogeneous product. This final product, now infused with 2.145 × 10^8^ CFU/mL, underwent quality testing to ensure an immediate post-formulation minimum recovery of ≥1 × 10^8^ CFU/mL following the method outlined in the subsequent section. The final product was then transferred to 50 mL conical Falcon tubes. For long-term storage, the conical Falcon tubes were snap-frozen by immersing in a mixture of ethanol and dry ice, then stored at −20 °C.

### Probiotic bacteria survival rate quantifications

To assess probiotic survival rates in PIMD, quadruplicate samples were analyzed with results expressed as mean ± standard deviation (SD). For liquid samples, one mL aliquots were used directly, while surface samples were collected using TRANSWAB^®^ for aerobes and anaerobes (Medical Wire & Equipment Co. Ltd.) and vortexed in one mL of supplemented BHI broth. The BHI broth was supplemented with filter-sterilized catalase (25 U/mL) and Isovitox (0.01 mL/mL; Roche Diagnostics Saudi Arabia LLC, Saudi Arabia) to neutralize hydrogen peroxide accumulation, which is particularly critical for maintaining viability of oxygen-sensitive *Bifidobacterium* species (Kawasaki et al., 2006). Samples underwent serial ten-fold decimal dilutions in identically supplemented BHI broth. From each dilution, 50 µL aliquots were plated in duplicate onto chocolate agar plates containing equivalent concentrations of catalase and Isovitox, then spread uniformly using sterile glass spreaders. Plates were incubated under microaerophilic conditions (5% O_2_, 95% N_2_,) at 37 °C for 48 h—parameters optimized to support growth of all PIMD constituent strains (Kawasaki et al., 2006; Couvert et al., 2023). Following incubation, colonies were enumerated and counts within the 30–100 CFU range were converted to log_10_ CFU/mL.

### Pathogen survival rate quantifications

Survival testing of indicator pathogenic bacteria on surfaces was performed in quadruplicate, with four independent replicates per experimental condition and selective medium. Sterile contact plates (Qingdao Hope Bio-Technology Co., Ltd., Shandong, China) containing selective media supplemented with lecithin (0.7 g/L) and Tween 80 (5 g/L) as neutralizing agents (Sozzi et al., 2019). The following media and incubation conditions were applied: (1) For the selective enumeration of *Staphylococcus aureus*, Mannitol Salt Agar (MSA) was used and incubated at 37  °C in ambient air. (2) To enumerate Enterobacteriaceae, including *Escherichia coli*, MacConkey Agar was employed and incubated at 37 °C under anaerobic conditions to inhibit the growth of aerobic *Pseudomonas* spp. (3) For the selective recovery of *Pseudomonas aeruginosa*, Pseudomonas Isolation Agar (PIA) was used, with incubation at 41 °C in ambient air. The neutralizing combination of lecithin (0.7 g/L) and Tween 80 (5 g/L) was empirically validated to quench both PIMD constituents and residual sodium hypochlorite, ensuring accurate pathogen recovery. Validation involved exposure–neutralization cycles using standardized bacterial suspensions, followed by enumeration on selective media. The neutralized samples demonstrated recovery of viable colonies, with no statistically significant difference (*p* > 0.05) in CFU counts compared to untreated controls. This confirmed the complete neutralization of any carry-over bactericidal activity during plating and validated the method’s suitability for reliable post-disinfection microbial enumeration.

### Storage stability assessment

PIMD stability was assessed by monitoring viable CFU/mL across under three storage conditions (−20 °C, 4 °C, 24 °C) and time intervals (four batches per temperature). Prior to testing, each bottle was homogenized (4 min gentle mixing; after being thawed for −20  °C samples), followed by serial dilution in supplemented BHI broth and culturing as previously described to determine probiotic survival rates.

### High-density polyethylene cutting boards samples

New high-density polyethylene (HDPE) cutting boards (handong HUAAO Engineering Technology Co., Ltd., China) were sectioned into 1 cm^3^ pieces using the BD Trek™ Powered Bone Biopsy System (Becton, Dickinson and Company, USA), sterilized by autoclaving and stored in sterile containers until used.

### PIMD probiotic survival rate on cutting boards samples

The upper surfaces of 124 HDPE cutting board pieces were aseptically loaded with 100 µL/cm^2^ of PIMD (contains ≥1 × 10^7^ CFU dose), placed in sterile petri dishes, and incubated at 24 °C. The probiotic viability measured immediately after application (Day 0) served as the internal time-zero control, establishing the baseline survival rate. The water activity (a_w_) of the resulting film was determined using Decagon Lite (AquaLab, USA). Notably, PIMD formed a sticky glossy moist film residue on the treated surfaces, indicative of the effective application of PIMD. Subsequently, four pieces were immediately retrieved on day 0, and another four pieces were collected every subsequent day for 30 days. Each piece was then placed in five mL of catalase- and Isovitox-supplemented BHI broth (described above), vortexed for 10 s, and serially diluted and cultured to quantify the probiotic bacteria, following the methodology described above.

### Ecological modeling of probiotic–pathogen dynamics

To simulate and optimize ecological interactions within the PIMD microbead system, two complementary modeling approaches were employed:

(1) Deterministic framework

A generalized Lotka–Volterra model (Qian, Lan & Venturelli, 2021; van den Berg et al., 2022) was adapted to quantify interspecies dynamics *dP*_*i*_/*dt* and *dP*_*k*_/*dt* that are the core of the differential equations. They represent the instantaneous rate of change of the population of probiotic (indexed *i*, *j*) and pathogens (indexed *k*, *m*) respectively, over time as seen in the Lotka-Volterra equations: between probiotics *i* and pathogens *k*:

\begin{eqnarray*}d{P}_{i}/dt& ={r}_{i}\cdot {P}_{i}\cdot (1-{\Sigma }_{j}{\alpha }_{ij}\cdot {P}_{j}/{K}_{i})-{\Sigma }_{k}{\beta }_{ik}\cdot {P}_{i}\cdot {P}_{k} \end{eqnarray*}

\begin{eqnarray*}d{P}_{k}/dt& ={r}_{k}\cdot {P}_{k}\cdot (1-{\Sigma }_{m}{\gamma }_{km}\cdot {P}_{m}/{K}_{k})-{\Sigma }_{i}{\delta }_{ki}\cdot {P}_{k}\cdot {P}_{i} \end{eqnarray*}

where, *r* denotes growth rates, *K* carrying capacities (CFU units) and *r*_*i*_ and *K*_*i*_ represent strain-specific growth rates and carrying capacities, respectively, while interaction coefficients (*α*, *β*, *γ*, *δ*) were derived from *in vitro* coculture assays. Parameter optimization was performed using Python (SciPy v1.8.0) with experimentally validated initial conditions employing nonlinear least-squares optimization (least_squares) and bootstrap validation (*n* = 1,000). Parameter ranges were as follow:

–Growth rates (*r*): 0.05–1.5/hour.

–Carrying capacities (*K*): 1 ×10^8^ CFU/cm^2^ or 1 ×10^9^ CFU/mL

–Interaction coefficients (*α*, *γ*): [−1.0, 1.0]

–Antagonism coefficients (*β*, *δ*): [0, 2.0]

–Integration window: 0–48 h (liquid) or up to 21 days (surface models).

(2) Stochastic Agent-Based Model (ABM)

A spatially explicit 2D agent-based model (ABM) was implemented in NetLogo 6.3.0 to simulate microbial community dynamics, incorporating four agent types: (i) Type A (*Bacillus* spp.) exhibiting lipopeptide secretion, (ii) Type B (*Lactobacillus* spp.) producing organic acids, (iii) Type C (*Bifidobacterium* spp.) with oxygen-sensitive metabolism, and (iv) Type P pathogens (*S. aureus*, *E. coli*). The model encoded two key ecological interactions: cross-feeding (Type B→Type C *via* short-chain fatty acids) and antagonism (Type A→Type P *via* bacteriocins). Simulations (500 iterations ≈ 24 h; *n* = 10 replicates per condition) generated three primary outputs: (a) time-series population dynamics, (b) spatial dominance heatmaps, and (c) pathogen exclusion probabilities.

Key processes per timestep (∼3 min real time):

–Growth ∝ (r × local nutrient × O_2_ × hydration factor)

–Secretion: Type A → M1 (lipopeptide); Type B → M2 (acid)

–Cross-feeding: Type C benefits from M2 (Hill function)

–Antagonism: pathogen mortality if M1/M2 >  threshold (p_*k*_ = 0.2/timestep)

–Biofilm transition: when ≥20 agents occupy a patch

Simulation conditions:

–10 stochastic replicates per condition

–500 iterations ≈ 24 h

–Diffusion (D_*m*_): 1 ×10^−6^ cm^2^/s (range: 10^−7^–10^−5^)

–Sensitivity analyses performed on all secretion and inhibition parameters

Outputs:

–gLV: population trajectories, extinction time, coefficients (*α*, *β*, *γ*, *δ*)

–ABM: spatial dominance heatmaps, pathogen exclusion probability, inhibition halo morphology.

### Simultaneous antagonism

A BHI agar plate was uniformly spread-plated with 100 µL of an indicator pathogen inoculant (prepared as described above) using a sterile glass spreader. This confluent pathogen lawn served as the negative control, confirming organism viability and that any observed antagonism was due to the PIMD formulations. Immediately afterward, a sterile toothpick tip was dipped into different PIMD formulations and stabbed onto the plate. The plate was then incubated at 37 °C for 12 h under 5% CO_2_ in air. Following incubation, the agar surface was examined for both biofilm competitive exclusion patterns and zones of inhibition. In the layout, the topmost stab inoculum represented the complete PIMD formulation, while three columns of three stab inocula were positioned directly beneath it: the first column contained probiotics alone, the second contained PIMD lacking anaerobes, and the third contained PIMD lacking aerobes. This arrangement enabled direct comparison of the full formulation with its partial combinations, with outcomes assessed on the basis of both biofilm exclusion and antimicrobial halo formation. The entire experiment was independently replicated four times to ensure reproducibility.

### Sink drains experiment

Sterilized stainless steel sink strainers (11.43 cm diameter; Shandong Huaye Co., Shandong, China) were used in quadruplet sets of three per bacterial species (*E. coli, P. aeruginosa,* or *S. aureus*), totaling twelve strainers per trial. Each strainer was contaminated with five µL bacterial inoculum at 41 defined grid points, arranged in a concentric radial pattern (five rings spaced one cm apart with eight radial spokes and a central point), totaling 205 µL per strainer. After gravity-assisted spreading (15–30° incline) and 1-hour incubation at ambient conditions (25 °C, ∼50% RH), strainers were treated with 205 µL of one of the following: (1) the PIMD test article; (2) the positive control (0.4% sodium hypochlorite); or (3) the negative control (probiotic-free microbeads impregnated with sterile BHI, lacking probiotic inoculum). Treatments were applied using the same grid pattern as for inoculation. Finally, a sterile, single-use polyurethane brush (five mm diameter; Jiangsu GuardKing Medical Equipment Co., Ltd.) was used to deliver 10 unidirectional strokes per strainer under controlled pressure. Thirty minutes and one-hour post-application, culturing was performed using contact plates (for pathogens) and TRANSWAB^®^ (for probiotics) as described above, with results expressed as percentage mean log_10_ reduction ± SD. An unpaired, two-tailed Student’s *t*-test was used to compare each disinfectant treatment (PIMD and the positive control) with the negative control, as well as to compare PIMD directly with the positive control, for each pathogen at both the 30-minute and 1-hour intervals. A *p*-value of <0.05 was considered statistically significant.

### Outdoor tiles contaminated with feces from non-human primates experiment

To evaluate PIMD’s efficacy in challenging outdoor environments, 50 × 50 cm ceramic tiles (Saudi Ceramic Co., Riyadh, Saudi Arabia) were sterilized by autoclaving and artificially contaminated with fresh fecal matter from Hamadryas baboons (*Papio hamadryas*) known to be contaminated with both *S. aureus* and Enterobacteriaceae, obtained from southwestern Saudi Arabia as described previously (Alqumber, 2024), and homogenized in sterile phosphate-buffered saline (PBS) to create a 10% w/v fecal slurry. Next, a calibrated grid pattern (10 × 10 squares, 1 cm^2^ each) was applied to each tile, with 100 µL of fecal slurry deposited per square. Tiles were air-dried in a biosafety cabinet (25 °C, 50% humidity, 1 h). After drying, disinfectant treatments were applied using the same grid-based spotting technique and volume as used for the fecal slurry. Treatment groups included: (1) PIMD; (2) the positive control (0.4% sodium hypochlorite); and (3) the negative control (probiotic-free microbeads). All treatments were applied at 100 µL per square, ensuring consistent coverage across all test surfaces. Bacterial viability following treatment was evaluated in quadruplicate, using selective culture-based enumeration as described previously.

### Pathogen exclusion test

To assess the sustained efficacy of the developed Probiotic-Infused Microbead Disinfectant (PIMD), a 50 µL/cm^2^ thin film was uniformly applied to 90 HDPE cutting board samples using a glass spreader. Subsequently, these treated boards were positioned in indoor air at 25 °C to simulate real-world conditions. Each day, sets of five treated samples were consecutively challenge-inoculated with indicator pathogens (10 µL/cm^2^ for each pathogen), namely *Staphylococcus aureus* isolate 1, *Escherichia coli* isolate 1, *Pseudomonas aeruginosa* isolate 1, or *Campylobacter jejuni* isolate 1. The inoculation, carried out with a glass spreader, ensured even distribution. Additionally, in separate sets of five tests, only one of the specified indicator pathogens was utilized. In these instances, the glass spreader was employed to dispense 50 µL/cm^2^, consisting solely of the single-pathogen indicator. Subsequent to inoculation, the board samples were left exposed to indoor air at 25 °C for an additional 180 min. Following this, tested surfaces were used to inoculate, by pressing firmly onto selective media as described above. This test evaluated the sustained activity of the PIMD biofilm. The internal/time-zero control was the disinfection capacity measured immediately after PIMD application, against which the sustained efficacy over 21 days was compared.

### *In vitro* antimicrobial activity testing

The extended spectrum of antimicrobial activity of the produced PIMD was evaluated through a spot assay. Initially, 100 µL inoculum of indicator pathogen was uniformly spread-plated with sterile glass spreader, creating a confluent lawn that served as the negative control. The undisturbed bacterial growth across the plate confirms the viability of the pathogen and the adequacy of the culture conditions. Any zone of inhibition that forms around the PIMD spot is therefore unequivocally attributable to the antimicrobial activity of the formulation and not to a failure of the pathogen to grow. Subsequently, 30 µL of the PIMD disinfectant was spotted on top of the inoculated culture. After incubation, the diameter of any inhibition zone formed around the PIMD spot was measured to determine the effectiveness of the disinfectant. Additionally, the diameter of the PIMD protective biofilm was measured. To distinguish bactericidal from bacteriostatic effects, material from within the inhibition zone was aseptically sampled and subcultured into fresh BHI broth. Bactericidal activity was confirmed only when no turbidity developed in BHI after 24 h of incubation and no colonies emerged on the original agar plate within the inhibition zone after an additional 24 h of incubation. Any growth detected under either condition was classified as bacteriostatic.

### Ethical, biosafety, and source approvals

Multi-drug-resistant isolates and Hamadryas baboon (*Papio hamadryas*) fecal samples, utilized to prepare the challenge inocula, were obtained under the permit of the Pathogen Surveillance Program (PSP; Project Number MOE-BU-1-2020), Deputyship for Research and Innovation, Al-Baha University, and the Ministry of Education, Saudi Arabia. Samples were transported using a triple-packaging system, comprising a primary water-tight 50 mL Falcon conical tube, sealed within a zip-lock plastic bag, and placed inside a rigid, insulated outer packaging of corrugated polyurethane foam. All experimental procedures involving the fecal inocula, multi-drug-resistant clinical isolates, and other potentially infectious materials were conducted in accordance with the protocols approved under the aforementioned permit. All procedures involving tile contamination and treatment were conducted by personnel wearing appropriate personal protective equipment (PPE), including laboratory coats, nitrile gloves, and safety goggles. Following experimentation, all test tiles, consumables, and liquid waste were inactivated by complete submersion in a mixture of 7.5% (w/v) povidone-iodine and 5% (w/v) chlorhexidine for 30 min, followed by autoclaving at 121 °C for 60 min prior to disposal.

## Results

### Consistency and stability of the PIMD during storage

**Figure 2:**
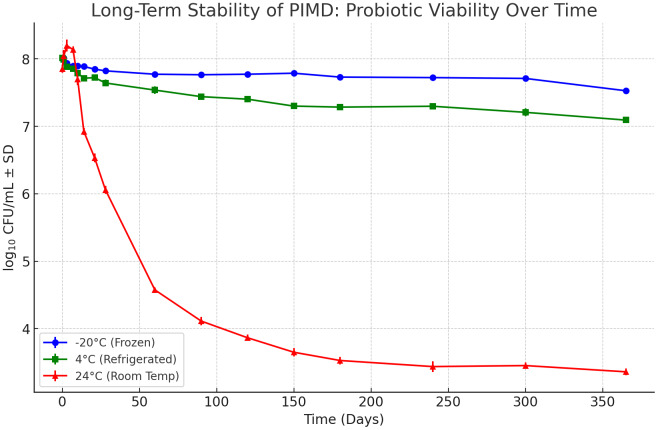
Storage stability of the Probiotic-Infused Microbead Disinfectant (PIMD). Viable probiotic counts (log_10_ CFU/mL) in PIMD formulations stored at −20 °C, 4 °C, and 24 °C (room temperature) over 365 days. Data points represent mean values ± standard deviation (*n* = 4 batches). Storage at −20 °C and 4 °C maintained high viability for ≥ 6 months. The initial increase at 24 °C suggests cell proliferation in the nutrient-rich matrix, followed by a sharp decline.

The PIMD formulation maintained a viscous, sauce-like consistency under all tested conditions, with water activity (a_w_) remaining stable between 0.5 and 0.7 throughout ambient storage. At day 0, viable counts across all storage conditions showed consistent recovery, with log_10_ CFU/mL values ranging from 7.54 to 8.00, reflecting the initial post-formulation viability threshold (≥1 × 10^8^ CFU/mL) and partial bacterial entrapment within the microbead matrix. Probiotic viability at −20 °C remained highly stable, with only a 0.46 log_10_ CFU/mL reduction after 365 days, indicating excellent cryoprotection. Storage at 4 °C showed a slightly greater decline, with a cumulative reduction of 0.89 log_10_ CFU/mL after one year, largely attributable to the gradual loss of oxygen-sensitive strains such as *Bifidobacterium* spp. and *Lactobacillus* spp. In contrast, samples stored at 24 °C exhibited a biphasic trend: an initial increase in log_10_ CFU/mL during the first 7 days—consistent with cell proliferation in a nutrient-rich matrix—followed by a sharp decline beginning on day 10. By day 365, a pronounced total reduction of 4.51 log_10_ CFU/mL was observed in the ambient storage group (Fig. 2).

### PIMD probiotic survival rate on HDPE cutting board samples

**Figure 3:**
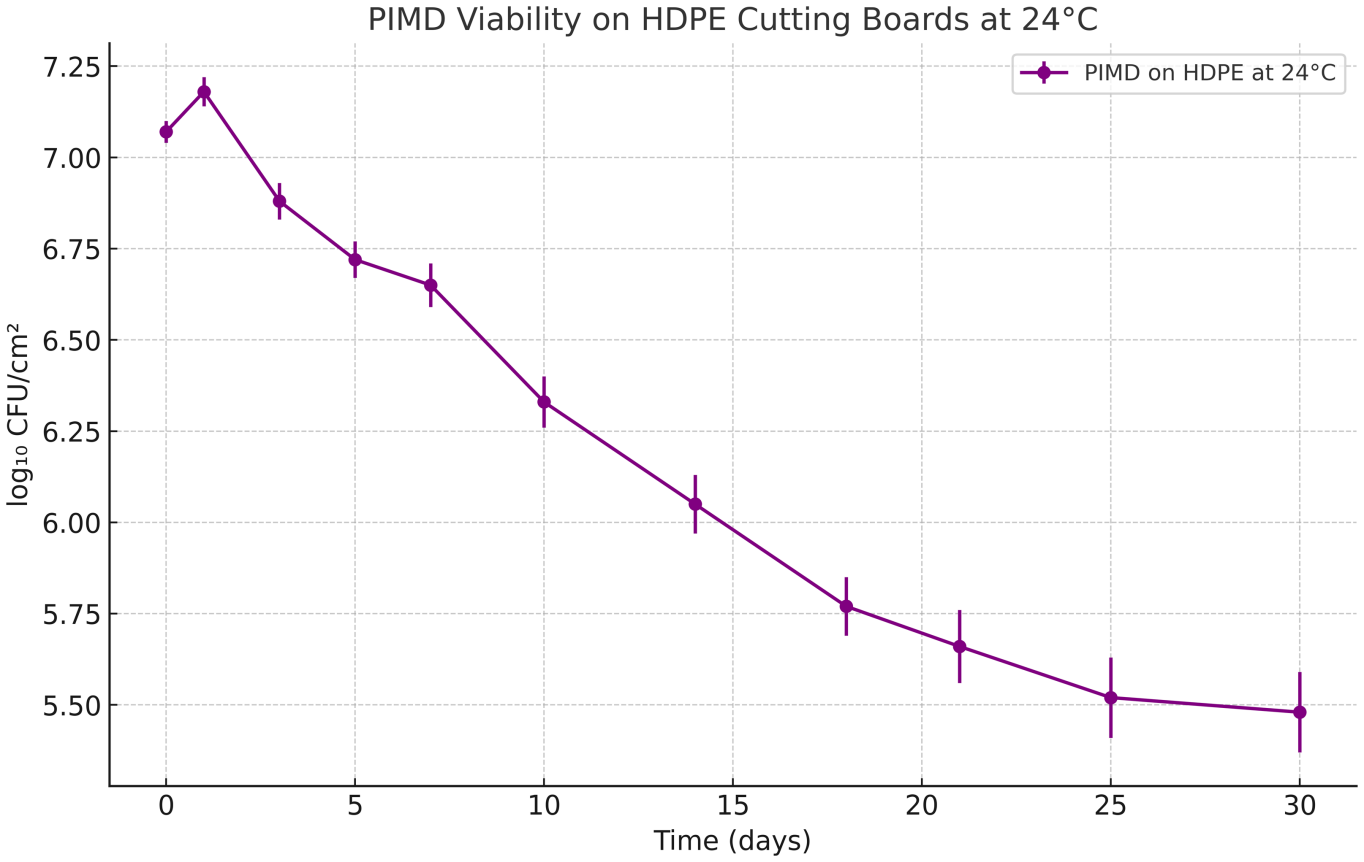
Probiotic survival and biofilm formation on high-density polyethylene (HDPE) cutting boards. Viable probiotic counts (log_10_ CFU/cm^2^) recovered from HDPE surfaces treated with PIMD and incubated at 24 °C over 30 days. Data points represent mean values ± standard deviation (*n* = 4 samples per time point). The initial increase at 24 h indicates active biofilm formation, followed by a gradual decline while maintaining a protective probiotic population (>5 log_10_ CFU/cm^2^) for the duration of the study.

Upon application of PIMD to HDPE cutting board pieces and incubation at 24 °C, the day 0 recovery was 7.07 ± 0.03 log_10_ CFU/cm^2^, consistent with expected surface recovery from the ≥10^7^ CFU/cm^2^ dose. Over the next 24 h, a modest increase to 7.18 ± 0.04 log_10_ CFU/cm^2^ was observed. Afterwards, viability gradually declined over the 30-day period. By day 7, CFU levels had dropped to 6.65 ± 0.06 log_10_ CFU/cm^2^, and by day 14 to 6.05 ± 0.08, despite stable water activity values (a_w_ 0.55–0.66) that would support moisture retention. At day 30, final counts reached 5.48 ± 0.11 log_10_ CFU/cm^2^, marking an approximate 1.6 log_10_ CFU/cm^2^ decline from baseline (Fig. 3).

### Ecological modeling results

The Lotka–Volterra simulations revealed that stable coexistence was not possible under the experimental parameterization: in all conditions, probiotics drove pathogens to extinction over time (the time-to-extinction ranged from 1 to 6.5 h and was dependent on the initial inoculum ratio), consistent with the Competitive Exclusion Principle (Stone et al., 2020; Gause, 1934). The highest inhibitory coefficients (*δ*_*ki*_) were observed for *B. pumilus* strain 33 and *L. plantarum* SF9C, with inferred per-capita killing effects (*β*_*ik*_) of 0.67 ± 0.09 and 0.59 ± 0.06, respectively. When simulated with multiple probiotic species, synergistic dynamics amplified suppression, reducing time-to-extinction for pathogens from 6.5 h to 1 h. The agent-based model supported these findings, demonstrating spatially segregated zones where oxygen-sensitive *Bifidobacterium* strains flourished near *Lactobacillus* clusters, indicating successful cross-feeding. Pathogens were excluded in 100% of simulations within 300–400 steps (simulated 12–18 h), with biofilm dominance established by probiotic agents. Notably, agents representing *Bacillus* species frequently colonized the periphery, forming inhibition “halos” that protected interior probiotic populations dominated by *Lactobacillus*–*Bifidobacterium* clusters, mirroring observed surface inhibition patterns.

### Simultaneous antagonism

**Figure 4:**
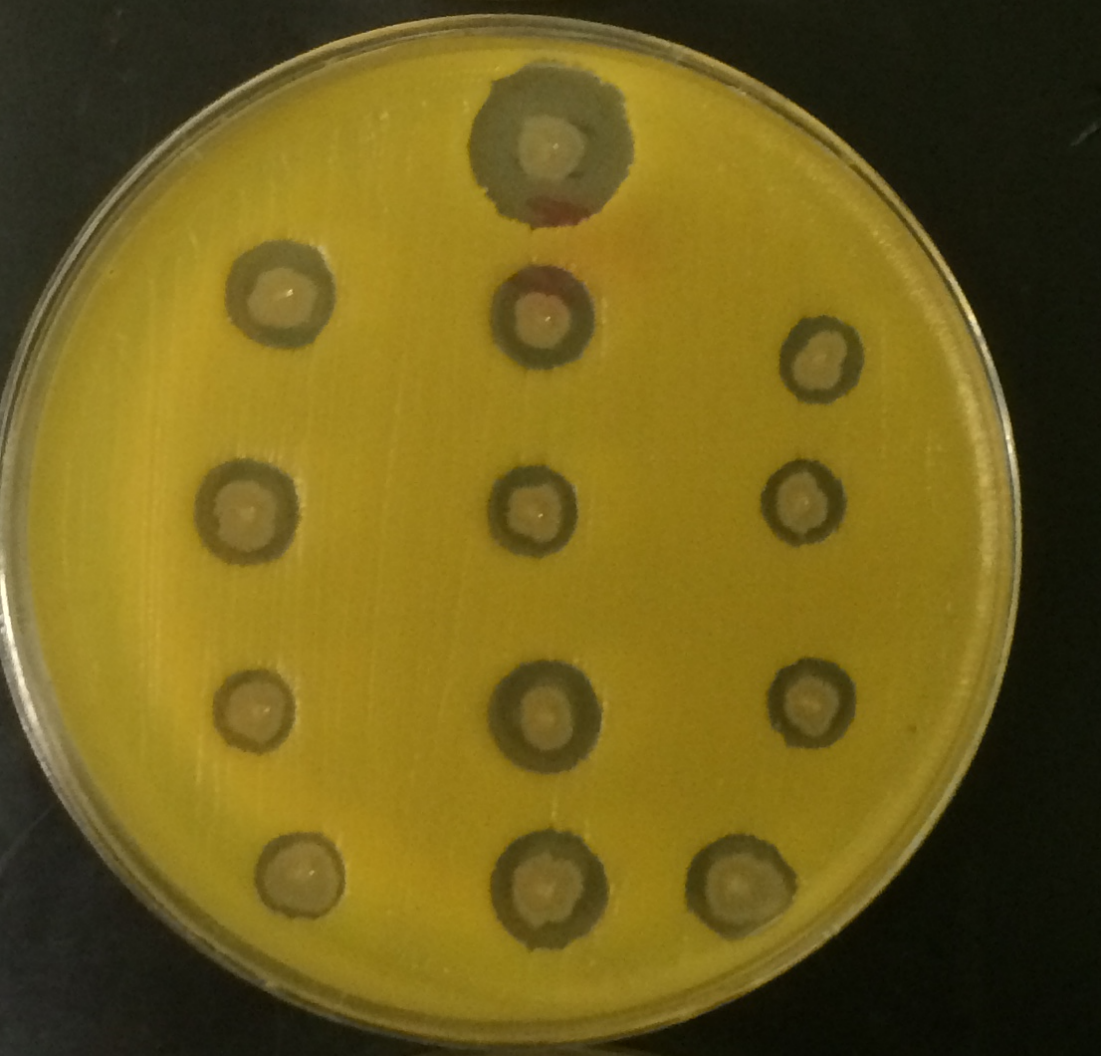
Simultaneous antagonism assay of the Probiotic-Infused Microbead Disinfectant (PIMD). A brain–heart infusion (BHI) agar plate was spread-plated with 100 μL of *Micrococcus luteus* strain T-18 and inoculated by toothpick stabs containing different PIMD formulations. The topmost stab represented the complete PIMD formulation (probiotics + activated charcoal + hydroxyapatite + carrier matrix). Directly beneath it, three columns of three stabs each were arranged to test partial combinations: column 1 contained probiotics alone, column 2 contained PIMD lacking anaerobes, and column 3 contained PIMD lacking aerobes. Following 12 h of incubation at 37  °C under 5% CO_2_, two distinct antagonistic effects were observed: outward probiotic biofilm expansion (competitive exclusion) and concentric inhibition zones (antimicrobial secretion). The complete PIMD formulation produced the most pronounced inhibition zone, confirming synergistic efficacy. Experiments were independently replicated four times with consistent results.

The PIMD demonstrated a clear dual antagonistic effect. First, probiotic biofilms extended outward from the toothpick inoculation points, with a mean spread diameter of 3.5 ± 1.5 mm, indicating effective competitive exclusion of the indicator pathogen. Second, concentric inhibition zones, ranging from 1 to 3.5 mm in width, were consistently observed, reflecting strong antimicrobial secretion by the probiotic consortium. These antagonistic effects were evident across all tested formulations, with the complete PIMD formulation producing the most pronounced inhibition halos (Fig. 4).

### Sink drains experiment results

The sink basin trials validated the rapid and sustained efficacy of PIMD in a high-risk, high-moisture surface environment. As shown in Tables 1 and 2, PIMD achieved significant pathogen reductions across all three tested species—*S. aureus, E. coli,* and *P. aeruginosa*. By 24 h post-treatment, pathogen loads were reduced below detectable levels. Importantly, PIMD sustained probiotic viability *in situ*, with stable colonization evident up to 21 days post-application with CFU counts ≥5.0 ± 0.2 log_10_ CFU/cm^2^.

**Table 1:** Sink drain log_10_ reductions and percentage efficacy after 30 min. Pathogen log_10_ reduction (mean ± SD, *n* = 4) achieved by PIMD and 0.4% sodium hypochlorite (positive control) compared to a negative control (sterile microbeads) on stainless steel sink strainers. PIMD achieved significant reductions against all three pathogens within 30 min (*p* < 0.05 *vs.* negative control).

Pathogen	Treatment	Percentage mean log_10_ reduction	Mean log_10_ reduction ± SD	*p*-value (*vs.* negative control)	*p*-value (*vs.* sodium hypochlorite)
*S. aureus*	PIMD	99.65%	2.46 ± 0.28	<0.001	0.063
*S. aureus*	Sodium hypochlorite	99.77%	2.63 ± 0.19	<0.001	
*S. aureus*	Negative Control	12.90%	0.06 ± 0.06		<0.001
*E. coli*	PIMD	99.54%	2.34 ± 0.22	<0.001	0.027
*E. coli*	Sodium hypochlorite	99.73%	2.57 ± 0.25	<0.001	
*E. coli*	Negative Control	8.8%	0.04 ± 0.05		<0.001
*P. aeruginosa*	PIMD	98.94%	1.98 ± 0.31	<0.001	0.041
*P. aeruginosa*	Sodium hypochlorite	99.12%	2.05 ± 0.27	<0.001	
*P. aeruginosa*	Negative Control	6.68%	0.03 ± 0.03		<0.001

**Notes.**

Data represent mean log_10_ reduction ± SD (*n* = 4); NaOCl: 0.4% sodium hypochlorite (positive control); Control: probiotic-free microbeads with sterile BHI (negative control); Statistical significance determined by unpaired two-tailed *t*-test (*p* < 0.001 *vs.* negative control); Calculation: a log reduction (LR) is converted to percentage reduction (PR) using the formula: PR = (1 − 10^−LR^) × 100.

**Table 2:** Sink drain log_10_ reductions and percentage efficacy after 1 h. Pathogen log_10_ reduction (mean ± SD, *n* = 4) achieved by PIMD and 0.4% sodium hypochlorite. By 1 h, PIMD achieved complete disinfection (≥99.9% reduction) of all pathogens, with performance comparable to the chemical disinfectant (*p* > 0.05 for PIMD *vs.* hypochlorite for each pathogen).

Pathogen	Treatment	Percentage Mean Log_10_ reduction	Mean Log_10_ Reduction ± SD	*p*-value (*vs.* negative control)	*p*-value (*vs.* sodium hypochlorite)
*S. aureus*	PIMD	99.991%	4.05 ± 0.24	<0.001	0.865
*S. aureus*	NaOCl	99.994%	4.22 ± 0.18	<0.001	
*S. aureus*	Control	18.717%	0.09 ± 0.07		
*E. coli*	PIMD	99.980%	3.70 ± 0.22	<0.001	0.735
*E. coli*	NaOCl	99.992%	4.12 ± 0.20	<0.001	
*E. coli*	Control	12.904%	0.06 ± 0.05		
*P. aeruginosa*	PIMD	99.971%	3.54 ± 0.26	<0.001	0.835
*P. aeruginosa*	NaOCl	99.981%	3.72 ± 0.23	<0.001	
*P. aeruginosa*	Control	8.799%	0.04 ± 0.04		

**Notes.**

Data represent mean log_10_ reduction ± SD (*n* = 4); NaOCl: 0.4% sodium hypochlorite (positive control); Control: probiotic-free microbeads with sterile BHI (negative control); Statistical significance determined by unpaired two-tailed *t*-test (*p* < 0.001 *vs.* negative control); Calculation: a log reduction (LR) is converted to percentage reduction (PR) using the formula: PR = (1 − 10^−LR^) × 100.

### Outdoor tile disinfection results

To assess performance under outdoor conditions with high organic burden, PIMD was evaluated on ceramic tiles contaminated with fecal slurry from *Papio hamadryas*, known to harbor *S. aureus* and Enterobacteriaceae (Alqumber, 2024). Initial pathogen loads averaged 7.65 ± 0.21 log_10_ CFU/cm^2^. Application of PIMD led to a 97.86 ± 0.42% reduction within 45 min. Complete disinfection (≤99.9% reduction) was achieved by 1 h. Sodium hypochlorite achieved complete disinfection (≤99.9% reduction) of pathogens within 45 min, whereas negative controls showed no significant reduction. Probiotic persistence was confirmed on PIMD-treated tiles, with viable probiotic recovery at ≥5.2 ± 0.31 log_10_ CFU/cm^2^ after 21 days. No probiotic recovery was observed in control groups.

### Pathogen exclusion test

PIMD successfully eliminated all indicator pathogens (*Staphylococcus aureus* isolate 1, *Escherichia coli* isolate 1, *Pseudomonas aeruginosa* isolate 1, and *Campylobacter jejuni* isolate 1) from the treated cutting boards in all testing conditions, within 1 h in ambient air. Disinfection abilities did not change in older boards (tested up to 21 days post-PIMD application). After 8 days, the colony forms were found to be dominated with *B. pumilus* strain 33 with traces of other probiotic strains.

### *In vitro* antimicrobial activity testing

The disinfectant demonstrated potent inhibitory effects against a broad spectrum of pathogenic bacteria, with *in vitro* spot assays revealing complete inhibition across all tested strains (Table 3). Post-exposure subculturing confirmed bactericidal activity against 34.9% of isolates (no viable growth), while the remainder exhibited bacteriostatic effects. The mean inhibition zone diameter measured was 13.84 ± 1.23 (*n* = 43) (Table 3).

**Table 3:** *In vitro* antimicrobial activity of PIMD against pathogenic bacteria.

**Strain specification**	**Inhibition diameter (mm)**	**Subculture result**
*Micrococcus luteus* strain T-18	15	Positive
*Streptococcus pyogenes* strain FF22	14	Positive
*S. anginosus* strain T-29	13	Negative
*S. pyogenes* strain 70-679	15	Negative
*S. uberis* strain T-6	14	Negative
*S. pyogenes* strain 71-679	15	Positive
*S. pyogenes* strain 71-698	16	Positive
*S. pyogenes* strain W-1	14	Negative
*S. equisimilis* strain T-148	15	Negative
*Enterococcus faecalis* strain 96	16	Positive
*E. faecalis* strain 97	13	Negative
*E. faecalis* strain 98	12	Positive
*S. dysgalactiae* strain 60	14	Positive
*S. dysgalactiae* strain 61	15	Positive
*S. dysgalactiae* strain 62	16	Positive
*S. agalactiae* strain 76	16	Positive
*S. agalactiae* strain 77	14	Positive
*S. agalactiae* strain 78	15	Positive
*Enterococcus faecalis* strain E024	14	Positive
*E. faecalis* strain C2	13	Negative
*E. faecalis* strain K13b	13	Positive
*Enterococcus faecium* strain TE1	12	Positive
*E. faecium* strain A10	14	Negative
*E. faecium* strain 5A9	13	Positive
*E. faecalis* strain JM95	13	Positive
*E. faecalis* strain JM101	12	Positive
*E. faecalis* strain JM112	13	Positive
*Salmonella enterica* subsp. *enterica* serovar Typhi isolate 1	15	Positive
*Salmonella enterica* subsp. *enterica* serovar Typhi isolate 2	13	Negative
*Escherichia coli* O157 isolate 1	14	Positive
Enteropathogenic *E. coli* isolate 1	12	Positive
Enteroaggregative *E. coli* isolate 1	12	Positive
*Campylobacter jejuni* isolate 1	13	Positive
*Listeria monocytogenes* isolate 1	14	Negative
Methicillin-resistant *Staphylococcus aureus* (MRSA) isolate 1	16	Positive
Methicillin-resistant *Staphylococcus aureus* (MRSA) isolate 2	15	Negative
Methicillin-resistant *Staphylococcus aureus* (MRSA) isolate 3	14	Positive
*Shigella dysenteriae* isolate 1	14	Positive
*Shigella flexneri* isolate 1	13	Positive
*Vibrio cholerae* isolate 1	13	Negative
*Vibrio parahaemolyticus* isolate 1	13	Negative
Carbapenem-resistant *Pseudomonas aeruginosa* (CRPA) isolate 1	12	Negative
Carbapenem-resistant *Pseudomonas aeruginosa* (CRPA) isolate 2	13	Negative

## Discussion

This study introduced a PIMD, engineered to protect and deliver a 12-strain probiotic consortium within a functional microbead matrix. Each microbead compartmentalizes either spore-forming probiotic strains (*e.g.*, *Bacillus* spp.) or non-spore-forming strains (*e.g.*, *Lactobacillus*, *Bifidobacterium*), providing isolated microenvironments that sustain strain-specific metabolic activity, such as oxygen gradients, pH, and metabolite production. This design supports the proliferation of different strains while minimizing inter-strain competition. Over time, strains optimally suited to the application environment (*e.g.*, aerobic surfaces for *Bacillus*, anaerobic niches for *Bifidobacterium*) can selectively expand, ensuring sustained pathogen exclusion.

PIMD is designed to combine immediate antimicrobial activity, mediated by probiotic-derived bioactive compounds like bacteriocins, lipopeptides and organic acids, with long-term competitive exclusion through the formation of a stable probiotic biofilm. Empirical validation *via* simultaneous antagonism assays confirmed two concurrent outcomes: (i) the establishment of a protective probiotic biofilm and (ii) formation of a distinct inhibition zone (Fig. 4), demonstrating PIMD’s capacity for both immediate pathogen eradication and sustained prevention of recolonization. This dual mechanism addresses a key limitation of conventional disinfectants, which often disrupt surface microbiomes and can lead to pathogen resurgence (Jing et al., 2020; Maillard & Pascoe, 2024). The carrier matrix, containing prebiotics, hemicellulose oligosaccharides, electrolytes, and nutrients in a saline solution, contributes to a waxy, hygroscopic biofilm that maintains stable water activity a_w_ between 0.5 to 0.7, creating a hydrated microenvironment conducive to prolonged probiotic survival and activity. The stability of hydration was confirmed by the consistent a_w_ levels, remaining stable within the range of 0.5 to 0.7 a_w_ *via* the potential adsorption and absorption of water from air and the carrier matrix contains prebiotics, hemicellulose oligosaccharides, electrolytes, ions, and other nutrients, all mixed in a normal saline solution.

The ecological modeling provided strong theoretical support for PIMD’s dual-functionality. The generalized Lotka–Volterra (gLV) models predicted rapid pathogen decline in the presence of multi-strain probiotic competition and antimicrobial action, affirming Gause’s Law (competitive exclusion principle) (Stone et al., 2020; Gause, 1934) and aligning with experimental disinfection outcomes. Agent-based models (ABMs) added a spatial dimension, illustrating how the microbead architecture facilitates ecological segregation and metabolic cooperation s—such as oxygen consumption by aerobes creating niches for oxygen-sensitive species. The emergence of synergistic biofilm clusters and peripheral antimicrobial “barriers” in these simulations paralleled the observed sustained probiotic viability and pathogen suppression on surfaces like high-density polyethylene (HDPE) cutting boards and sink drains. Simultaneous antagonism results matched the modeling results, and both confirmed PIMD’s immediate pathogen clearance, colonization and biofilm formation. The gLV model offers a robust, quantitative fit of bulk population dynamics and interaction strengths, while the ABM elucidates how localized, stochastic interactions and spatial structure drive emergent community patterns, such as biofilm formation and inhibition halo morphology, which are inaccessible to the deterministic framework. Future improvements to the model could incorporate real-time metabolomics data, and *in situ* microfluidic studies may validate diffusion patterns observed in simulations.

Stability assessment confirmed that PIMD maintained a logarithmic survival percentage above 75% for up to a year when stored at −20 °C, underscoring its robust shelf-life. On HDPE cutting boards, PIMD demonstrated high efficacy, achieving pathogen reduction comparable to sodium hypochlorite against clinically relevant organisms like *Staphylococcus aureus, Escherichia coli, Pseudomonas aeruginosa, and Campylobacter jejuni*. *In vitro* spot assays confirmed PIMD’s broad-spectrum efficacy against Gram-positive bacteria (*e.g.*, *Listeria monocytogenes, Clostridioides difficile, S. aureus, Enterococcus* spp.), and Gram-negative (*e.g.*, *E. coli* O157, *C. jejuni*) pathogens (Table 3). Subculturing from inhibition zone margins demonstrated a bactericidal outcome (no growth on selective media) against 34.9% of tested strains, with bacteriostatic effects observed in the remainder. The pathogen exclusion tests on cutting boards demonstrated prolonged efficacy for over 21 days post-application, supported by the sustained viability of probiotics on HDPE surfaces for over 30 days.

The observed rapid antimicrobial efficacy (*e.g.*, ≥99.9% pathogen reduction within 1 h) is attributed to the synergistic action of antimicrobial metabolites produced by specific probiotic strains. For instance, *Bacillus pumilus* strain 33 and *Bacillus licheniformis* B63 are documented producers of broad-spectrum bacteriocins and lipopeptides (Al-Qumber & Tagg, 2006; Hou et al., 2025), while *Lactobacillus* spp. (*e.g.*, *L. plantarum* SF9C) secrete plantaricin, organic acids and hydrogen peroxide (Butorac et al., 2020). This immediate suppression reduces the pathogen load, creating conditions conducive for the surviving probiotics to establish a biofilm that allows for long-term competitive exclusion (Stone et al., 2020; Tomé et al., 2023; Moon, Seo & Kwon, 2025). Once the initial pathogen load is reduced, the surviving probiotics establish a biofilm that perpetuates Gause’s Law by outcompeting residual pathogens for resources and adhesion sites (Gause, 1934; Qian, Lan & Venturelli, 2021; Van den Berg et al., 2022). Thus, PIMD operates through a sequential dual mechanism: rapid kill followed by ecological dominance. The intrinsic antibiotic-resistance of probiotic strains (Campedelli et al., 2018; Gueimonde et al., 2013) further supports their sustained viability and enhances their capacity to outcompete antibiotic-resistant pathogens through ecological exclusion rather than relying on conventional antibiotic mechanisms (Aghamohammad & Rohani, 2023; Jain et al., 2023). Future studies should quantify the relative contributions of antimicrobial metabolites *versus* competitive exclusion to guide the rational optimization of the probiotic consortium and carrier matrix for specific applications.

The composition of PIMD was formulated to ensure probiotic viability, robust surface adherence, and safety for potential indirect food-contact surfaces. Each excipient serves a specific functional role: Dimethyl sulfoxide (DMSO) acts as a cryoprotectant (Aarattuthodi et al., 2025), with evidence indicating its safety in small doses (Kollerup Madsen et al., 2018). Lecithin and polysorbate function as emulsifiers, enhancing dispersion and promoting stable adhesion to hydrophobic surfaces (Ziar & Riazi, 2022; Zhao et al., 2023). A lipid blend (olive oil, coconut oil, mutton tallow) forms a structural matrix with antioxidants that helps sustain viability and creates a semi-occlusive film to slow desiccation (Culmone et al., 2025; Jadhav & Annapure, 2023; Junkuszew et al., 2020). Muscone is included solely as an odor-masking agent, with studies indicating its biocompatibility and low toxicity at practical exposure levels (Wang et al., 2025).

In practical terms, if probiotic biofilms fail to establish, native environmental microbes—including opportunistic or pathogenic species—will inevitably recolonize these moist substrates such as sinks and drains, often with greater risk and resistance to antimicrobial agents than probiotic strains. The PIMD system, therefore, aims to maintain a controlled, beneficial microbiota rather than sterility. Nonetheless, whenever indicated—such as in environments involving immunocompromised individuals, critical care settings, or surgical areas—routine cleaning and disinfection with conventional methods remains essential to eliminate both non-probiotic and probiotic. This periodic intervention ensures that microbial balance is preserved and mitigates the risk of overgrowth, horizontal gene transfer, or persistence of unintended strains—risks that are substantially higher in conventional, non-probiotic microbial communities. Moreover, because PIMD is synthesized from well-characterized, biosafety-certified probiotic strains, it is inherently designed to reduce rather than increase such risks.

## Conclusions

This study demonstrates that the spatially engineered Probiotic-Infused Microbead Disinfectant (PIMD) delivers both immediate and sustained antimicrobial action. Rapid pathogen suppression results was demonstrated, and prolonged probiotic colonization was achieved, maintaining efficacy for over 21 days. These findings contribute to a growing body of knowledge supporting PIMD as a novel disinfectant, offering a strategy that maintains microbial equilibrium, reduces reliance on harmful chemicals, and mitigates antimicrobial resistance (Stone et al., 2020; Gause, 1934; Tomé et al., 2023). Its application on food and high-contact surfaces may enhance hygiene while promoting ecological safety. Limitations of our study include testing on a limited range of surfaces, and the lack of quantification of the relative contributions of metabolites *versus* competitive exclusion and strain-specific effects. Future research should explore more real-world surface applications focusing on large-scale validation across a wider range of real-world surfaces and environmental conditions, quantifying the relative contributions of metabolites *versus* competitive exclusion, and further investigating strain-specific effects.

##  Supplemental Information

10.7717/peerj.20803/supp-1 Supplemental Information 1 Raw data for Fig. 1

10.7717/peerj.20803/supp-2 Supplemental Information 2 Dataset for Fig. 2

10.7717/peerj.20803/supp-3 Supplemental Information 3 Raw data for Tables 1 and 2

## Additional Information and Declarations

### Data Availability

The following information was supplied regarding data availability: The raw data are available in the Supplemental Files.
